# Texture Evolution and Nanohardness in Cu-Nb Composite Wires

**DOI:** 10.3390/ma14185294

**Published:** 2021-09-14

**Authors:** Shihua Xiang, Xiaofang Yang, Yanxiang Liang, Lu Wang

**Affiliations:** 1 Shenyang National Laboratory for Materials Science, Chongqing University, Chongqing 400044, China; xiangsh@cqu.edu.cn (S.X.); liangyx@cqu.edu.cn (Y.L.); wanglu@cqu.edu.cn (L.W.); 2 International Joint Laboratory for Light Alloys (Ministry of Education), Department of Materials Science and Engineering, Chongqing University, Chongqing 400044, China

**Keywords:** Cu-Nb composite, texture, dynamic recrystallization, nanoindentation, interface

## Abstract

Multifilamentary microcomposite copper-niobium (Cu-Nb) wires were fabricated by a series of accumulative drawing and bonding steps (ADB). The texture of the Cu matrix in these wires was studied using electron backscattered diffraction (EBSD) and transmission electron microscopy (TEM). Dynamic recrystallization during cold drawing caused a weakening of the <111> texture in the micron-scale Cu matrix at high values of true strain. A sharp <111> texture was observed in the nano-scale Cu matrix due to the suppression of dynamic recrystallization. The grain size was reduced by the higher level of dynamic recrystallization at high strains. The relation between the nanoindentation behavior of the different Cu matrix and the grain sizes, Cu-Nb interface, and texture was established.

## 1. Introduction

Over the past two decades, Cu-Nb microcomposites have been widely used in defense, aerospace, and magnetic applications for their excellent combination of mechanical properties, high conductivity, and thermal stability [1,2,3,4]. Several techniques have been used to fabricate these materials, including accumulative drawing and bonding (ADB) [5,6], melt and deform [7,8], accumulative roll bonding (ARB) [9,10,11], and magnetron sputtering [12,13]. Compared with other techniques, the ADB process enables the production of Cu-Nb microcomposite wires more than 100 m in length [14]. During this process, a multi-scale Cu matrix with grain sizes ranging from a few microns to a few nanometers is formed [15]. Metals experiencing axisymmetric deformation after ADB will develop a fiber texture and only one direction is needed to fully represent the preferred orientation [16,17]. The large number of embedded Nb fibers affects the deformation and texture development of the Cu matrix [18]. A variety of constraints, including the Cu-Nb interface area and the matrix dimensions, will alter the deformation compared to non-composite FCC materials [19]. It is well established that the texture exerts a strong influence on the mechanical properties and electrical conductivity of such materials. For example, wires with a <111> fiber texture will exhibit higher stiffness than wire with a random orientation [20,21,22]. Thus, studies on texture evolution are required to better understand the process–structure relationships.

The microstructure and texture evolution of Cu-Nb microcomposites have been widely investigated [6,8,19,23,24,25,26]. Using X-ray diffraction (XRD) and neutron diffraction, researchers [6,23,25] observed that a sharp <111> fiber texture with a weak <100> texture for the Cu matrix and a distinct <110> texture for Nb developed in these composite wires. The intensities of these two fiber texture components in the Cu matrix are intricately affected by intermediate annealing and deformation strain, whereas the texture of Nb is basically unchanged [23,24]. Popova et al. [25] found that both <111> and <100> texture components are weakened at higher cold drawing strains due to dynamic recovery and recrystallization in Cu-Nb composite wires. However, it is impossible to characterize the texture at different scales in the Cu matrix by XRD. Several studies [19,26] utilized TEM to investigate the crystallographic relationship between the Cu matrix and Nb fibers. Deng et al. [19] reported that a comparatively parallel relationship of <111> Cu and <110> Nb with a few degrees of deviation formed in Cu-Nb wires at a strain of 24.8. Nevertheless, the texture obtained by TEM did not support this result statistically. Recently, an EBSD investigation of as-deformed Cu-Nb wires [15,16] at different scales demonstrated different proportions of <111> and <100> texture [15] within regions of the Cu matrix. However, these studies mainly focused on the micron-scale texture of the Cu matrix rather than the nanoscale. It seems that a complete understanding of texture evolution for Cu-Nb microcomposite wires during deformation requires further systematic research.

ADB Cu-Nb microcomposite wires are well known as a bi-phase material with a multi-scale microstructure. Conventional mechanical testing methods are not well suited to studying the mechanical properties of Cu channels at different scales. Nanoindentation testing has been used extensively for multi-scale and dual-phase materials to analyze the mechanical properties with high spatial resolution [27,28,29,30,31,32]. Using nanoindentation, Thilly et al. [30] found that Cu-Nb interfaces have a strong blocking effect on dislocation slip and attributed the high strength of Cu-Nb wires to a size effect and interface strengthening. Nanoindentation was applied along the rolling (RD) and transverse (TD) directions of an ARB Cu-Nb nanolaminate to study the effect of anisotropy [32]. Unfortunately, there are few nanoindentation studies focused directly on the Cu matrix in composite wires at different strains. 

In this paper, the evolution of each layer of the Cu channel was characterized in detail at different levels of strain. The principles of texture formation at the different scales of the Cu matrix are discussed. Nanoindentation testing was applied to quantify the hardness values for different Cu channels. The influential effects of the Cu matrix, grain sizes, Cu-Nb interface, and texture on the mechanical properties of the microcomposite wires were established.

## 2. Materials and Methods

High-purity niobium (99.9%) and oxygen-free high-conductivity (OFHC) copper were utilized to fabricate Cu-Nb composite wires with a fixed number of Nb fibers using an ADB process. The ADB process results in a multi-scale Cu matrix containing continuous parallel Nb fibers with a maximum number of up to 85^4^ (N = 85^4^, determined by the number of drawing steps) and with diameters varying in the range of 10 nm to 500 μm. Four different types of specimens were prepared after each pass of the first four ADB process, with N = 85, 85^2^, 85^3^, and 85^4^. These samples were designated by the number of Nb fibers. The ADB process can be seen in detail elsewhere [3].

EBSD specimens were cut from the ADB wires, and electron polished in a solution of 83.3% H_3_PO_4_/16.7% H_2_O at a voltage of 2 V (<0.01 A) and 20 °C. The EBSD characterization was carried out in a scanning electron microscope (TESCAN MIRA 3, Tescan Corporation, Brno, Czech Republic) with step sizes from 0.01 to 1.5 μm. TEM samples were prepared by a low-temperature ion-thinning technique and examined in an FEI Tecnai F20 electron microscope (Thermo Fisher Scientific, Waltham, MA, USA) at a voltage of 200 kV.

Nanoindentation tests were performed on each channel of copper in different samples using a Hysitron Triboindenter (Hysitron Inc., Minneapolis, MN, USA) and a Berkovich indenter. A peak load of 6 mN and loading time of 5 s were applied with loading and unloading rates of 12 mN/min. The positions of indentation were selected using an optical microscope (OM) equipped in Triboindenter. Atomic force microscopy (AFM, Hysitron Inc., Minneapolis, MN, USA) was performed to identify the surface profile of the post-test indents. More than 30 tests were carried out in each test area to get accurate nanohardness values. The indentation regions were observed via SEM to determine the area fraction of each phase. All the indentation tests were carried out in the longitudinal (drawing) direction.

## 3. Results

### 3.1. Microstructure and Texture

**Figure 1:**
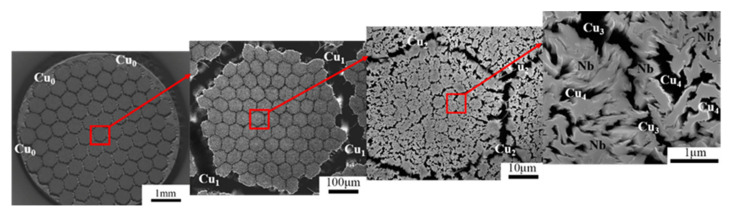
SEM micrograph showing the multi-scale microstructure of N = 85^4^ Cu-Nb microcomposite wires. In the maximum magnification view, the Nb fibers exhibit light contrast embedded in a dark-contrast Cu matrix (removed by etch).

The cross-sectional SEM microstructure of the Cu-Nb composite wire containing 85^4^ of Nb fibers is shown in Figure 1. The 85^4^ Nb fibers were embedded in a Cu matrix with five different spacings of Cu_n_: from the largest outer-most jacket (*n* = 0) to the finest nanoscale Cu (*n* = 4). Similarly, the 85^3^ Nb fibers were embedded in a Cu matrix with four spacings of Cu_n_ (from Cu_0_ to Cu_3_). Similar results were also observed in the 85^2^ and 85 samples. Based on the distance between Nb fibers, the scale of the copper matrix could be divided into three categories: micro-scale copper (M-Cu, larger than 1 μm), sub-micron-scale copper (S-Cu, 100 nm–1 μm), and nanoscale copper (N-Cu, less than 100 nm). The dimensional parameters and characteristics of the Cu-Nb composite wires at different ADB stages are displayed in Table 1. A linear intercept method was used to measure the dimension of the Cu_i_ and Nb fibers based on the SEM and TEM images (only *d*_Cu3_, *d*_Cu4_, and *d*_Nb_ in 85^4^).

**Figure 2:**
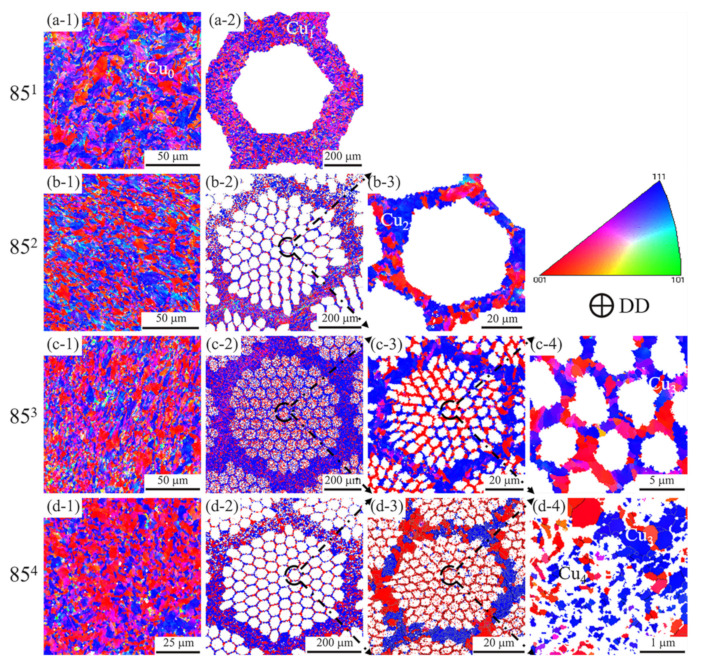
EBSD maps of copper matrix with different scales in different samples. (**a-1**) Cu_0_ in 85^1^; (**a-2**) Cu_1_ in 85^1^; (**b-1**) Cu_0_ in 85^2^; (**b-2**) Cu_1_ in 85^2^; (**b-3**) Cu_2_ in 85^2^; (**c-1**) Cu_0_ in 85^3^; (**c-2**) Cu_1_ in 85^3^; (**c-3**) Cu_2_ in 85^3^; (**c-4**) Cu_3_ in 85^3^; (**d-1**) Cu_0_ in 85^4^; (**d-2**) Cu_1_ in 85^4^; (**d-3**) Cu_2_ in 85^4^; (**d-4**) Cu_3_ and Cu_4_ in 85^4^.

EBSD maps (at different scales) of the Cu matrix in the four Cu-Nb wires are shown in Figure 2. A strong <111> and <100> fiber texture developed in the Cu matrix during the drawing deformation, a result consistent with those in the literature [23,24]. The total proportion of <100> and <111> texture increased gradually with the strain. There were grains with other orientations in the 85^2^ Cu_1_, yet it was nearly invisible in the other Cu channels (Cu_2_, Cu_3_ and Cu_4_). A more extensive substructure was observed in the 85^4^ Cu_2_ sample compared to 85^4^ Cu_3_. Notably, a higher fraction of substructure developed in the <111> grains than in the <100> grains.

**Figure 3:**
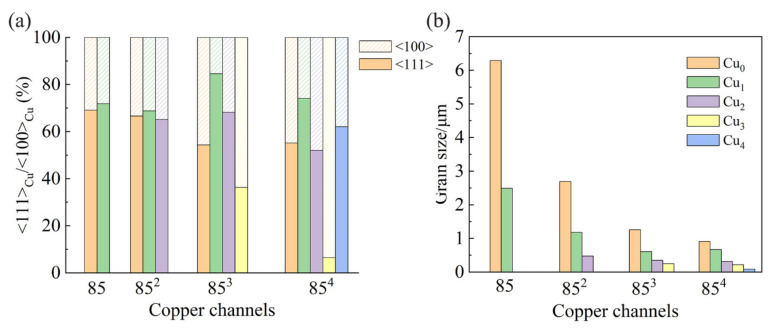
(**a**) Normalized area fraction of <111> and <100> texture in copper matrix, (**b**) grain size of copper matrix in different samples.

Figure 3a displays the normalized statistical results of the <111> and <100> texture components. A comparison between different samples with the same level of Cu at M-Cu (Cu_0_ and Cu_2_) shows that the <111> texture weakened generally with increasing true strain, whereas the <111> texture in Cu_1_ showed a contrary tendency. In each sample, the degree of deformation due to the ADB process increased from Cu_1_ to Cu_4_, and the <111> texture increased at first (from Cu_0_ to Cu_1_) and then decreased (from Cu_1_ to Cu_3_). In 85^4^ Cu_3_, the proportion of <111> texture decreased to less than 10%. In nanoscale Cu_4_, however, more than 60% of the grains were highly <111> textured. That is, the intensity of the <111> texture was enhanced at strains larger than 19.1, showing a contrary tendency compared to previous work by Popova et al. [25]. Thus, it is necessary to investigate the relation of drawing strain and the matrix size on the development of texture.

The grain sizes for the Cu channels in different samples are shown in Figure 3b. Severe dynamic recrystallization resulted in a weaker <111> texture and grain refinement. As the Cu channel size was reduced to a few hundred nanometers, the Cu-Nb interfaces began to play an important role. Single Cu grain filled the space between niobium fibers. This reduction in grain size decreased with the increase in dynamic recrystallization.

**Figure 4:**
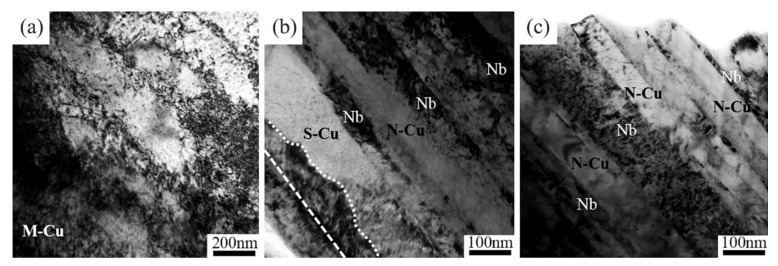
Bright field TEM images of 85^4^ sample (**a**) dislocation cells in M-Cu, (**b**) longitudinal sections showing Nb fibers surrounded by S-Cu and N-Cu, and (**c**) longitudinal sections showing clean N-Cu and Nb fibers.

Figure 4 shows the bright field images of copper regions in 85^4^. Large dislocation cells were observed in M-Cu, as shown in Figure 4a, indicating that dynamic recrystallization occurred in the Cu matrix at high deformation. The Nb fibers and Cu channels are marked in Figure 4b,c. There were significant differences in the interface where the Cu matrix width was more than 100 nm (S-Cu) or less than 100 nm (N-Cu), as shown in Figure 4b. Insignificant contrast induced by tangled dislocations was observed at the Cu-Nb interface where the Cu matrix width was less than 100 nm. The straight white dashed line marks the Cu-Nb interface in an area where a large number of dislocation tangles was observed.

### 3.2. Nanoindentation Tests

Given that the *d*_Cu3_ and *d*_Cu4_ in 85^3^ and 85^4^ samples were less than 3 μm, it was difficult for the indenter to cover only Cu_3_ or Cu_4_ zone. Therefore, only the nanohardness of Cu_i_ (i < 3) was directly tested in the present work. The nanohardness of Cu_3_ and Cu_4_ was calculated using a modified rule of mixtures (ROM) [30,33], taking the multi-scale nature of the composites into account:

(1) $$H_{\mathrm{com}}=X_{\mathrm{Nb}}H_{\mathrm{Nb}}+X_{\mathrm{Cu}3}H_{\mathrm{Cu}3}+X_{\mathrm{Cu}4}H_{\mathrm{Cu}4}$$

where *H*_com_ is the nanohardness of the tested regions and *X*_α_ is the volume fraction of the Nb or Cu_i_ (as seen in Table 2); the reference data for Nb crystals at micron(*H*_μ-Nb_) and nanometer scales (*H*_n-Nb_) were 2.4 and 4.2 GPa, respectively [30]. Therefore, for the 85^3^ sample:

(2) $$H_{\mathrm{cu}3}=\frac{H_{\mathrm{Cu}3+\mathrm{Nb}}-X_{\mu -\mathrm{Nb}}H_{\mu -\mathrm{Nb}}}{X_{\mathrm{Cu}3}}$$

As for the 85^4^ sample, where *d*_Cu3_, *d*_Cu4_ and *d*_Nb_ are in the nano-scale range, the *H*_Cu3_ and *H*_Cu4_ could be calculated:

(3) $$H_{\mathrm{cu}4}\;=\;\frac{H_{\mathrm{Cu}4+\mathrm{Nb}}-X_{n-\mathrm{Nb}}H_{n-\mathrm{Nb}}}{X_{\mathrm{Cu}4}}$$

(4) $$H_{\mathrm{cu}3}\;=\;\frac{H_{\mathrm{Cu}3+\mathrm{Cu}4+\mathrm{Nb}}-X_{\mathrm{Cu}4+\mathrm{Nb}}H_{\mathrm{Cu}4+\mathrm{Nb}}}{X_{\mathrm{Cu}3}}$$

**Figure 5:**
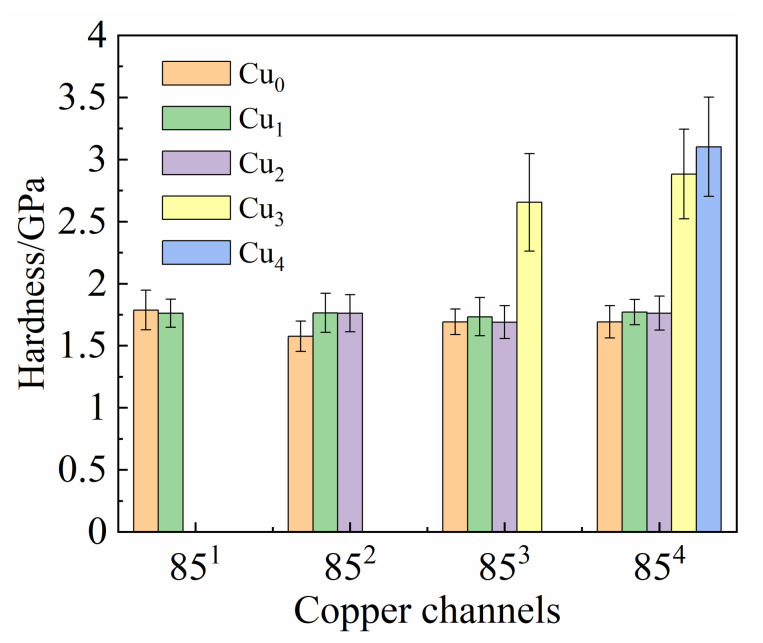
Nanohardness data of copper channels in different Cu-Nb wires.

The nanohardness of Cu regions in the different Cu-Nb wires is illustrated in Figure 5. The grain size decreased drastically with the Cu channel dimension from the Cu_0_ region of the 85 sample to the Cu_2_ region of the 85^4^ sample. However, no significant change of nanohardness was observed in any of the examined Cu_i_ (i < 3) regions. The average hardness was measured to be 1.72 ± 0.14 GPa, with a maximum value of 2.06 GPa and a minimum of 1.42 GPa. These results are lower than the experimental results from Thilly [30], but closer to the data in the literature [34]. The nanohardness of Cu_3_ in the 85^3^ sample was about 2.65 GPa, and the hardness of Cu_3_ and Cu_4_ in 85^4^ were 2.88 and 3.10 GPa, respectively. Compared with Cu_i_ (i < 3), the ultra-high hardness of Cu_3_ in 85^3^ and Cu_3_/Cu_4_ in 85^4^ indicate that there were other strengthening mechanisms in play.

**Figure 6:**
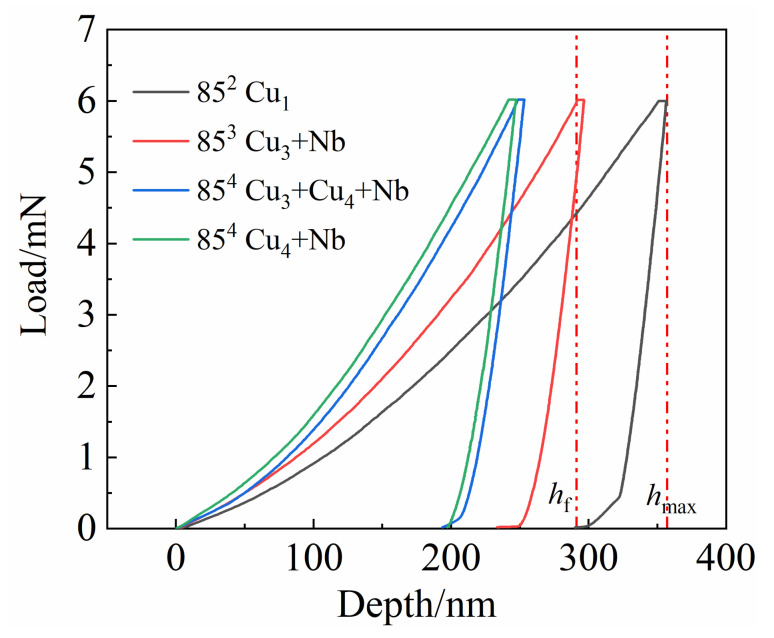
Typical load/displacement curves for different test areas of Cu-Nb composite wires.

**Figure 7:**
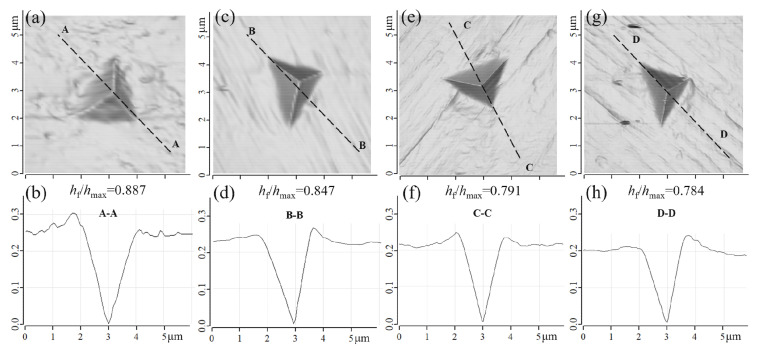
AFM images and corresponding surface profiles of indentations in different positions of Cu-Nb wires: (**a**,**b**) Cu_1_ channel in 85^2^; (**c**,**d**) Cu_3_+Nb region in 85^3^; (**e**,**f**) Cu_3_+Cu_4_+Nb region in 85^4^; (**g**,**h**) Cu_4_+Nb region in 85^4^.

The depth ratio after unloading and at peak load (*h*_f_/*h*_max_) is usually used to interpret the indentation behavior. Typical load/displacement curves of the examined samples are shown in Figure 6. The *h*_f_, *h*_max_, and other parameters are derived from these curves. Pharr et al. [35,36] concluded that when the *h*_f_/*h*_max_ ratio is less than 0.8, the pile-up can be ignored. That is, the nanohardness value is reliable when *h*_f_/*h*_max_ < 0.8. Figure 7 displays AFM images and surface profiles at four typical indentation regions. Obviously, only in the nanocomposite regions (Cu_3_+Cu_4_+Nb/Cu_4_+Nb regions in 85^4^) was the *h*_f_/*h*_max_ less than 0.8. Little pile-up was found in these regions (Figure 7f,h). In contrast, extended pile-up around the indent was observed in the regions with *h*_f_/*h*_max_ > 0.8 (Figure 7b,d).

## 4. Discussion

### 4.1. Texture Analysis

**Figure 8:**
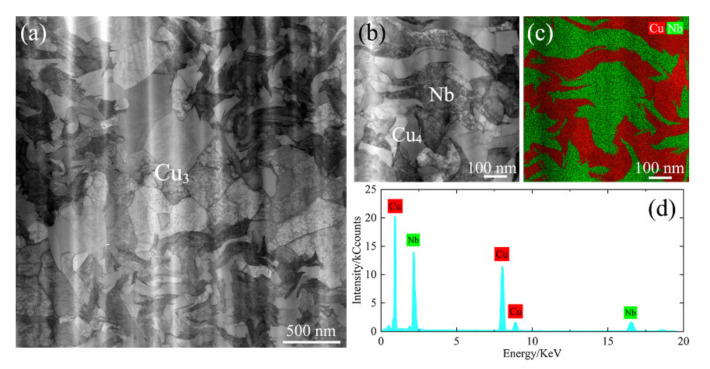
Transverse sectional HAADF-STEM of 85^4^ Cu-Nb microcomposite wire, (**a**) Cu_3_+Cu_4_+Nb region, (**b**) Cu_4_+Nb region, (**c**) EDS elemental mapping, and (**d**) EDS spectrum of (**b**).

The formation of <100> texture in micro-scale Cu during drawing is related to dynamic recovery and recrystallization, driven by deformation energy stored in the form of dislocations [37]. At low strain levels, dislocation pileups at grain boundaries or entangled dislocation substructures are developed. As the true strain increases, dislocations accumulate at low-angle sub-grain boundaries. High-angle grain boundaries are formed as the misorientation gradually increases. This mechanism is fully illustrated in the dynamic recrystallization process of M-Cu. Lee et al. [38] studied the effect of temperature on fiber texture, suggesting that recrystallization leads to the formation of <100> texture. Yang et al. [39] considered the effect of cold drawing on the microstructure evolution of Cu wires and found that dynamic recrystallization took place at strains greater than 1.91. In the present work, the smallest strain in the Cu channel was 4.7, much larger than 1.91. Dynamic recrystallization at this strain level led to a decrease in <111> texture and increase in <100> texture in M-Cu and S-Cu. As depicted in Figure 8a, the grains of Cu_3_ in 85^4^ exhibited a recrystallized morphology with a strong <100> texture. The less-developed substructure developed in the <100> grains of 85^4^ Cu_2_ also supports this interpretation.

As shown in Figure 3a, the proportion of <100> texture in the Cu_3_ channel in the 85^3^ sample was more than 60%. At higher deformation levels, the recrystallized <100> grains rotated to the <111> orientation [40], resulting in a stronger <111> texture. Meanwhile, the occurrence of dynamic recovery and recrystallization enhanced the <100> texture. After the last ADB step, the Cu_3_ in 85^3^ became Cu_4_ in 85^4^. Grain rotation was competed with dynamic recovery and recrystallization during the high-strain deformation.

Figure 8b presents the transverse sectional microstructure of Cu_4_+Nb in 85^4^. Compared with the equiaxed structure in Cu_3_ in 85^4^, most grains of Cu_4_ underwent severe deformation. Instead of Cu grain boundaries, a high density of Cu-Nb interfaces were created in the Cu_4_+Nb region. Such interfaces often play an important role in determining the properties of composite materials [1,19,41,42] and are especially important in composites with nanoscale microstructures, since a large number of defects are present [43,44]. Misfit dislocations and vacancies can act as obstacles for dislocation movement and are ideal sinks for dislocation annihilation [44]. When the Cu matrix size is reduced to submicron scale, the effect of the interface becomes more potent. Deng et al. [19] found a sharp increase in interface density within the nano-scale microstructure of a Cu-Nb composite. Figure 3a shows that the 85^4^ sample with both nanoscales and sub-micron scales of Cu_4_ exhibited a sharp <111> texture. In Figure 4b, the contrast induced by dislocation tangles in the Cu matrix was observed near the Cu-Nb interface where the Cu channel width was greater than 150 nm. A considerable amount of N-Cu in Cu_4_ was created due to the high strain of Cu_4_ (finest Cu channel) and the limitation of the Cu-Nb interfaces. Insignificant dislocation tangles were observed in the Cu channels when the width was close to or less than 100 nm (Figure 4c). Tangled dislocations can be eliminated by mutual annihilation or interface diffusion [45]. The deformation mechanism of the nanoscale Cu-Nb wire was confined to a layer slip that involved the movement of single dislocation loops on parallel planes between Nb fibers [46,47], which indicates that there was basically no dislocation entanglement in the nanoscale Cu matrix. Thus, dynamic recrystallization in these nanoscale Cu channels is more difficult due to the low driving force, and the deformation-induced <111> texture of the nanoscale Cu matrix was preserved.

### 4.2. Nanoindentation Behavior

Thilly et al. [30] reported that the averaged hardness of the largest Cu channels (*d*_Cu_ > 10 μm) is 2.17 GPa. This overestimated hardness is attributed to the pile-up around the indent. The pile-up can underestimate the calculated contact area between indenter and materials, leading to overestimation of the hardness values. In the present study, the h_f_/h_max_ was close to 0.88 in Cu_i_ (i < 3) channels, resulting in an underestimation of the calculated contact area and a positive deviation of the actual hardness. Although the pile-up phenomena could overestimate the hardness when the *h*_f_/*h*_max_ > 0.8, the change trend of hardness in different regions was not affected.

Microstructure dimension *d*, such as grain size and phase spacing, has a significant effect on the nanohardness [30,34,48]. Generally, the hardness is not dependent on size when *d* > 10 μm, whereas significant hardening occurs as *d* is reduced from 10 to 1 μm [30]. The *d*_Cu2_ in 85^3^ and 85^4^ composite wires was less than 10 μm. However, compared with other Cu channels with larger microstructure size, the hardness increase seemed unusually high in both samples. Li et al. [34] investigated the hardness of nano–micro-structured bulk copper by nanoindentation, and the results showed that the hardness values of coarse-grained Cu and nanocrystalline Cu were about 1.1 GPa and 2.1 GPa, respectively. The minimum grain size of Cu_i_ (i < 3) in the tested areas in the 85^4^ composite wire was about 0.5 μm due to dynamic recrystallization; thus, the hardness was close to that of coarse-grained Cu, inferring that nanohardness is similar regardless of the scale of the Cu matrix.

The calculated hardness values of Cu_3_ in 85^3^ and Cu_3_/Cu_4_ in 85^4^ were higher than *H*_Cui_ (i < 3). However, since the *h*_f_/*h*_max_ decreased with the size of the microstructure dimension, an overestimated hardness due to pile-up cannot explain the ultra-high hardness in these areas. For 85^3^, the grain size in Cu_3_ was less than 300 nm, leading to a positive deviation of the calculated hardness since the grain refinement strengthening. When the *d*_Nb_ was about 3 μm, Nb grains were nano-grains, resulting in an underestimation of *H*_μ-Nb_ calculated based on Equation (2). This is another possible explanation for the ultra-high hardness of Cu_3_ in 85^3^. For 85^4^, the *d*_α_ (α = Cu_3_, Cu_4_, Nb) was smaller than 1 μm, and there was little pile-up in the Cu_3_+Cu_4_+Nb/Cu_4_+Nb region. However, a large number of Cu-Nb interfaces existed with the potential for a large influence on the obvious hardness values [19,47]. Previous research has demonstrated that microhardness increases with the interface area density. Therefore, the unusually high nanohardness observed in this study was quite possibly a result of the interface density.

In addition, crystallographic orientation has a great effect on the hardness response in nanoindentation tests [27,28,29]. Wang et al. studied the mechanical properties of single-crystal Cu with different crystallographic orientations through nanoindentation, revealing that the nanohardness of (100)-, (110)-, and (111)-oriented single crystalline Cu are 1.33, 1.48, and 1.20 GPa, respectively [28]. Roa et al. studied the relationship between γ-(fcc) austenite grain orientation and nanoindentation hardness in metastable stainless steel, and found that (100) and (110) grains have lower hardness than (111) grains [27]. In the present study, the Cu_4_ channel in 85^4^ had strong <111> fiber texture compared to other Cu channels, which could also have resulted in the abnormally high level of hardness.

## 5. Conclusions

The texture and nanohardness of the different Cu channels in ADB Cu-Nb microcomposite wires were investigated. The main conclusions are as follows:

(1) Two fiber texture components, i.e., <111> and <100>, were found in the Cu matrix. In the micro-scale Cu matrix, the <111> texture weakened at higher levels of strain; for instance, the proportion of the <111> texture of Cu_3_ in 85^4^ was less than 10%. In submicron and nanoscale Cu channels, a sharp <111> texture was observed due to the suppression of dynamic recrystallization at the interface. The grain size was affected by dynamic recrystallization and decreased as the degree of deformation increased. The grain size was reduced by the higher level of dynamic recrystallization at high strains.
(2) The nanohardness of Cu_i_ (i < 3) in different Cu-Nb composite wires was similar, even though their grain sizes were orders of magnitude different. The slightly overestimated hardness value was caused by the pile-up near the indentation. There was no obvious size effect when *d*_Cui_ > 3 μm.
(3) Cu_3_ and Cu_4_ in 85^3^/85^4^ exhibited ultra-high nanohardness. For Cu_3_ in the 85^3^ sample, this result was mainly ascribed to the size effect and the underestimation of *H*_Nb_ in the calculation process. The size effect, high density of Cu-Nb interfaces, and texture effects were suggested for the unusually high nanohardness of Cu_3_ and Cu_4_ in the 85^4^ Cu-Nb composite wires.

## Figures and Tables

**Table 1:** Dimensional characteristics of the selection of Cu-Nb wires.

Sample	N	*d* (mm)	%Cu	*d*_Cu1_ (μm)	*d*_Cu2_ (μm)	*d*_Cu3_/μm	*d*_Cu4_ (nm)	*d*_Nb_ (μm)	η
85^1^	85	6.74	62	149 ± 15	–	–	–	428 ± 20	9.6
85^2^	85^2^	5.57 *	73	63 ± 14	14 ± 4	–	–	41 ± 8	14.4
85^3^	85^3^	5.64 *	79	63 ± 19	9 ± 2	2 ± 1	–	3 ± 1	19.1
85^4^	85^4^	5.06	84	60 ± 16	5 ± 2	462 ± 170 (nm)	97 ± 60	119 ± 90 (nm)	24.8
**Volume Fractions**									
85^3^ *X*_Nb_ = 0.21 *X*_Cu0_ = 0.222 *X*_Cu1_ = 0.225 *X*_Cu2_ = 0.124 *X*_Cu3_ = 0.219									
85^4^ *X*_Nb_ = 0.16 *X*_Cu0_ = 0.238 *X*_Cu1_ = 0.169 *X*_Cu2_ = 0.172 *X*_Cu3_ = 0.095 *X*_Cu4_ = 0.167									

Notes: N, the Nb filament number; d, the total diameter of the Cu-Nb wire (* hexagon); %Cu, the total volume fraction of Cu; *d*_Cui_, the measured width of the Cu_i_ channel; *d*_Nb_, the width of the Nb fibers; η, the total logarithmic true strain. (η = ln(A_0_/A), where A is the final cross-sectional area of the sample after deformation and A_0_ is the cross-sectional area of the wire before drawing). *X*α, the volume fraction of α.

**Table 2:** Volume fraction and nanohardness of the composite regions in 85^3^ and 85^4^ Cu-Nb wires.

Tested Regions	*X*_Cu3_	*X*_Cu4_	*X*_Nb_	*H* (GPa)
85^3^ Cu_3_+Nb	0.51	–	0.49	2.53 ± 0.19
85^4^ Cu_3_+Cu_4_+Nb	0.225	0.395	0.38	3.47 ± 0.16
85^4^ Cu_4_+Nb	–	0.51	0.49	3.64 ± 0.21

## Data Availability

Not applicable.
